# Impact of uPA/PAI-1 and disseminated cytokeratin-positive cells in breast cancer

**DOI:** 10.1186/s12885-019-5857-0

**Published:** 2019-07-15

**Authors:** Bruno Märkl, Martin Kazik, Nadia Harbeck, Elzbieta Jakubowicz, Reinhard Hoffmann, Thomas Jung, Dieter Steinfeld, Gerhard Schenkirsch, Günter Schlimok, Daniel Oruzio

**Affiliations:** 1 Institute of Pathology, Universitätsklinikum, Stenglinstraße 2, 86156 Augsburg, Germany; 2 Clinic for Anesthesiology and Intensive Care, Universitätsklinikum Augsburg, Augsburg, Germany; 3 Brustzentrum, Frauenklinik, Universität München (LMU), Munich, Germany; 4 Institute of Laboratory Medicine and Microbiology, Universitätsklinikum Augsburg, Augsburg, Germany; 5 Clinic for Gynecology and Obstetrics, Universitätsklinikum Augsburg, Augsburg, Germany; 6 Gynecology, Gemeinschaftspraxis Gynäkologische Onkologie, Augsburg, Germany; 7 Clinical and Population-based Cancer Registry of Augsburg, Augsburg, Germany; 8 Hematology and Oncology, Diakonissenkrankenhaus, Augsburg, Germany; 9 Onkologische Praxis MVZ, Rehling, Germany

**Keywords:** Breast cancer, Circulating tumor cells, Proteases, Prognosis

## Abstract

**Background:**

The protease uPA and its inhibitor PAI-1 play major roles in hemostasis and are also involved in cancer progression. This is mainly caused by their ability to degrade extracellular matrix-facilitating tumor cell migration. This study aimed to investigate the impact of uPA/PAI-1 and disseminated cytokeratin-positive cells (dCK+) on the outcome and the existence of synergistic effects.

**Methods:**

We retrospectively analyzed a cohort of 480 breast cancer cases with known uPA/PAI-1 and dCK+ status. uPA/PAI-1 was tested on fresh tumor samples using a commercial ELISA test. Bone marrow aspirates were investigated immunocytochemically for CK18.

**Results:**

DCK+ cells were identified in 23% of cases. uPA positivity was significantly associated with the occurrence of dCK+ cells (*P* = 0.028). uPA and PAI-1 were significantly associated with outcome in the subgroup of early-stage cases without chemotherapy. DCK+ cells alone were not prognostic. However, we found synergistic effects. In the subgroup of node-negative cases with and without chemotherapy, the prognostic impact of uPA and PAI-1 was enhanced in cases with additional dCK-positivity (triple +). In cases without chemotherapy, triple-positive status was independently prognostic (HR: 9.3 CI: 1.1–75) next to T stage.

**Conclusions:**

uPA and PAI-1 seem to influence the metastatic potential of dCK+ cells, which underlines its important role in tumor progression.

## Background

Breast cancer is the most common malignant tumor and the leading cause of cancer-related death in females in Germany [1]. Prognosis estimation and therapy stratification are mainly based on tumor grade, stage, hormone receptor status, HER2 status, and proliferation rate [2]. Next to those well-established prognostic and predictive markers, several new approaches have been pursued to further improve the prediction of outcome and therapy response in early breast cancer stages. Several multigene assays for fresh and formalin-fixed paraffin-embedded (FFPE) tissue samples have been developed and integrated into clinical practice [3]. The individual immune response has also gained increasing attention in breast cancer as it has in many other entities. Both outcome and response to chemotherapy are influenced by the microenvironment. Denkert al. showed that the extent of tumor-infiltrating lymphocytes is associated with the response to neoadjuvant chemotherapy [4]. Cytotoxic T-cells play an important role in tumor surveillance. The ability of tumors to escape from this surveillance is one of the hallmarks of cancer defined by Hanahan and Weinberg [5]. Matrix degradation is another way to facilitate tumor progression. In this context, the plasminogen/plasmin system has an important function. This system consists of several components, including uPA, which promotes the activation of plasminogen to plasmin, and the uPA-receptor (uPAR) as well as PAI-1 and PAI-2, which function as inhibitors of uPA. uPA, uPAR, and PAI-1 have been found to be important prognostic and predictive biomarkers of tumor progression. Next to their function in matrix degradation, several additional effects, such as activation of proliferation, anti-apoptosis, and angiogenesis have been discovered. This is very likely the reason for the adverse effect of PAI-1 that otherwise would be expected to be cancer suppressive. The prognostic relevance of the plasminogen/plasmin system has been reported in many cancer entities [6]. However, only in breast cancer has the evidence reached a sufficient level for general recommendations regarding its clinical use [7]. High levels of matrix-degrading proteases promote tumor cell migration and dissemination. Circulating tumor cells in the blood stream or disseminated cells in the bone marrow are believed to be the origin of distant metastases. Therefore, their detection can serve as biomarkers indicating an increased risk of disease progression. While testing of circulating tumor cells has not yet been introduced into the clinical routine, many studies have shown its clinical relevance in several cancer entities, including breast cancer. In the latter, it was shown to be prognostic and helpful in monitoring the response of adjuvant therapy [8]. Given the function of proteases in cancer, an association between the level of uPA and PAI-1 and the occurrence of disseminated tumor cells could be assumed. The aim of this retrospective study was to evaluate the relationship and potential interaction of the biomarkers uPA/PAI and disseminated cytokeratin-positive (dCK+) cells in the bone marrow.

## Methods

### Patients

Patients with breast cancer treated in the Klinikum Augsburg between 1999 and 2010 were included in this study. Bone marrow aspiration by puncture of both iliac bones was performed in all patients in the operating room right before lumpectomy or mastectomy, respectively. Informed and written consent for this procedure was obtained from all patients. Follow-up data were provided by the clinical and population-based cancer registry of Augsburg. Additional information, including data concerning adjuvant therapy, were obtained from the clinical files which were screened for each patient. Neoadjuvant chemotherapy was a criterion of exclusion. The study was approved by the internal review board of the Klinikum Augsburg. Raw data are available from an open repository.

### Immunocytochemical evaluation of circulating CK+ cells in the bone marrow

The samples were processed immediately after bone marrow aspiration. The technique has been described previously. In brief, the protocol for preparing the cytological samples was initially established for the detection of CK+ cells in bone marrow aspirates [9, 10]. The mononuclear cells were separated by Ficoll–Hypaque density gradient centrifugation (density, 1.077 g per mole) at 900×g for 30 min. The cells were then washed and centrifuged at 150×g for 5 min. Approximately 1 × 10^6^ cells were placed on each glass slide.

**Fig. 1:**
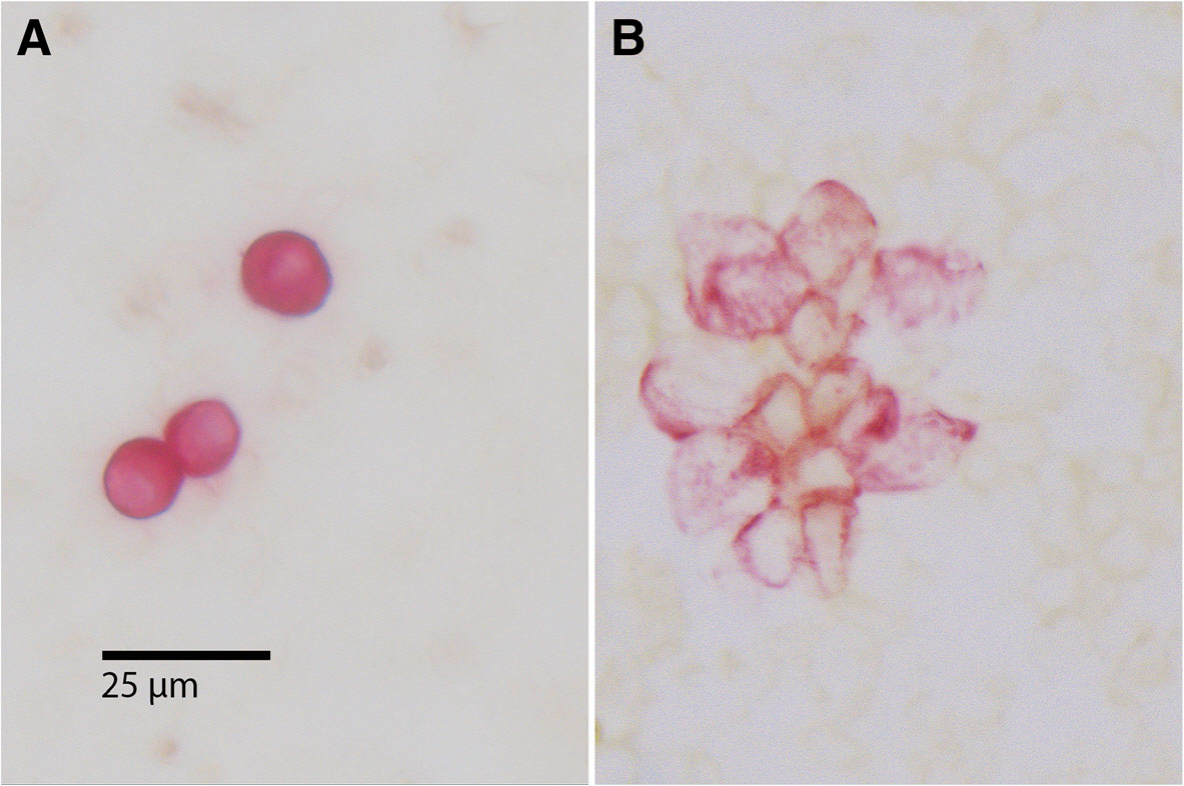
Immunocytochemically detected disseminated cells **a**) Three cytokeratin-positive (CK+) single cells; **b**) A cluster of 13 cytokeratin-positive cells. Note: These stainings were performed without counter-staining; therefore, the nuclei of the tumor cells are visible only as empty spaces within the cells, which explains different amounts of cytokeratin staining

To detect epithelial cells within the peripheral blood, a monoclonal antibody against cytokeratin 18 (Clone CK18 (Clone CK2), 1: 100; Chemicon, Hofheim, Germany) was used. The reactions were developed with the alkaline phosphatase anti–alkaline phosphatase (APAAP) technique combined with a new fuchsin stain to indicate antibody binding, as previously described [9, 10]. CK+ cells and clusters were counted manually (Fig. 1). All slides were screened by an experienced technician. All positive cases were confirmed by a hemato-oncologist (DO). Data concerning interobserver agreement between these two investigators are not available.

### uPA/PAI-1 ELISA testing

The lumpectomy or mastectomy specimens were brought to the laboratory of the pathology department immediately after resection without time delay. Representative samples were obtained from the fresh specimens after thinly slicing, based on macroscopic and palpatory examination. Attention was paid to avoid sampling areas with or near core biopsy defect. For the evaluation of the tissue levels of uPA/PAI-1, a commercial enzyme-linked immune sorbent assay (ELISA) test (Femtelle Test (EF 899), Sekisui Diagnostics, Stamford, CT) was used. The test was conducted according to the protocol recommended by the manufacturer and has been described previously [4]. In brief, frozen tissue samples were disrupted by mechanical force under permanent cooling. Tris-Buffer, supplemented with the non-ionic detergent Triton X-100, was used to extract the tumor cell cytosol. The suspensions were centrifuged to separate the soluble fractions from the cell debris. The total protein concentrations of the cytosolic fractions were measured. On day 2, a diluted tissue extract was added to antibody-coated microwells and incubated overnight. On day 3, detection antibodies were added and incubated. After another incubation step with the enzyme conjugates, the reactions were stopped with 0.5 M of H2SO4, and the absorption of the solution was measured using a microwell reader at 450 nm. The levels of uPA and PAI-1 were expressed in nanograms per milligram (ng/mg) of tumor protein. The cut-off-level for uPA and PAI-1 were 3 and 14 ng/mg protein, respectively, which are the same cut-offs routinely used in clinical settings [11]. Cases with values under the cut-offs were classified as negative and all others as positive.

### Statistical analysis

uPA and PAI values were categorized as *positive* and *negative* based on the defined cut-offs (3 and 14 ng/mg protein, respectively). All calculations were performed using those dichotomized values.

The Mann-Whitney rank sum test was used to compare numeric values. Correlations were calculated with Pearson Product Correlation. Tabulated data were compared using the chi-squared (χ^2^) test. For the survival analysis, Kaplan-Meier curves were calculated, and differences were analyzed with the log-rank test. The mean overall survival times were calculated because the median survival was not reached in most analyses. For the determination of the median follow-up time, the method of Schemper and Smith [12] was used. The Cox regression proportional hazards model was used for the multivariate analysis of cancer-specific analysis. All calculations were performed using the Sigma Plot 13.0 software package (Systat, Richmond, VA, USA). *P*-values < 0.05 were considered significant. For the survival analyses, we calculated additional thresholds considering the issue of multiple testing using the procedure of Bonferroni-Holm [13].

## Results

### Patients and correlations with clinico-pathological factors

The case characteristics are summarized in Table 1. A total of 480 patients primarily diagnosed and treated between 1999 and 2010 were included, with 80% of the cases from the years 2003 to 2006. The median follow-up time was 81 months (CI: 76–86 months). Presence of dCK+ cells was associated with the occurrence of lymph node metastases (*P* < 0.005). There was a trend toward a higher rate of dCK+ cells with increasing pT-stage (*P* = 0.100). uPA and PAI-1 levels were significantly associated with grade (*P* < 0.001 and *P* < 0.007) and progesterone receptor negativity (*P* = 0.01 and *P* = 0.023). uPA also showed an association with estrogen receptor negativity (*P* = 0.04), while PAI-1 showed only a trend in this direction (*P* = 0.06). Furthermore, a significant correlation with HER2 positivity was found for PAI-1 (*P* = 0.022).

**Table 1:** .

	Complete collection *n* = 480 (100%)	Positive dCK+ cells *n* = 110 (23%)	Negative dCK+ cells *n* = 370 (77%)	UPA pos. *n* = 220 (46%)	UPA neg *n* = 260 (54%)	PAI 1 pos. *n* = 326 (68%)	PAI 1 neg. *n* = 154 (32%)
Age							
< 51 years	115 (24%)	27 (25%)	88 (24%)	50 (23%)	65 (25%)	78 (24%)	37 (24%)
51–58 years	76 (16%)	19 (17%)	57 (15%)	39 (18%)	37 (14%)	50 (15%)	26 (17%)
59–66 years	129 (27%)	28 (25%)	101 (27%)	58 (26%)	71 (27%)	84 (26%)	45 (29%)
67–75 years	111 (23%)	25 (23%)	86 (23%)	51 (23%)	60 (23%)	78 (24%)	33 (21%)
> 75 years	49 (10%)	11 (10%)	38 (10%)	22 (10%)	27 (10%)	36 (11%)	13 (8%)
			*p = 0.9 n.s*		*p = 0.8 n.s.*		*p = 0.8 n.s.*
Tumor Size							
pT1	244 (51%)	52 (47%)	192 (52%)	116 (53%)	128 (49%)	166 (51%)	78 (51%)
pT2	191 (40%)	45 (41%)	146 (39%)	88 (40%)	103 (40%)	133 (41%)	58 (38%)
pT3	33 (7%)	11 (10%)	22 (6%)	11 (5%)	22 (8%)	20 (6%)	13 (8%)
pT4	11 (2%)	1 (1%)	10 (3%)	4 (2%)	7 (3%)	7 (2%)	4 (3%)
unknown	1 (0%)	1 (1%)	0 (0%)	1 (0%)	0 (0%)	0 (0%)	1 (1%)
			*p = 0.1 n.s.*		*p = 0.4 n.s.*		*p = 0.5 n.s.*
Grading							
G1	45 (9%)	11 (10%)	34 (9%)	14 (6%)	31 (12%)	31 (10%)	14 (9%)
G2	275 (57%)	61 (55%)	214 (58%)	111 (50%)	164 (63%)	172 (53%)	103 (67%)
G3	157 (33%)	37 (34%)	120 (32%)	94 (43%)	63 (24%)	122 (37%)	35 (23%)
unknown	3 (1%)	1 (1%)	2 (1%)	1 (0%)	2 (1%)	1 (0%)	2 (1%)
			*p = 0.9 n.s*		***p < 0.001 sign.***		***p < 0.007 sign.***
Nodal Status							
pN0	249 (52%)	53 (48%)	196 (53%)	112 (51%)	137 (53%)	171 (52%)	78 (51%)
pN1	148 (31%)	30 (27%)	118 (32%)	73 (33%)	75 (29%)	103 (32%)	45 (29%)
pN2	47 (10%)	10 (9%)	37 (10%)	22 (10%)	25 (10%)	29 (9%)	18 (12%)
pN3	29 (6%)	15 (14%)	14 (4%)	10 (5%)	19 (7%)	19 (6%)	10 (6%)
unknown	7 (1%)	2 (2%)	5 (1%)	3 (1%)	4 (2%)	4 (1%)	3 (2%)
			***p < 0.005 sign.***		*p = 0.6 n.s.*		*p = 0.8 n.s.*
UICC-stage							
stage I	147 (31%)	33 (30%)	114 (31%)	61 (28%)	86 (33%)	95 (29%)	52 (34%)
stage IIA	167 (35%)	30 (27%)	137 (37%)	84 (38%)	83 (32%)	121 (37%)	46 (30%)
stage IIB	68 (14%)	15 (14%)	53 (14%)	32 (15%)	36 (14%)	45 (14%)	23 (15%)
stage IIIA	46 (10%)	10 (9%)	36 (10%)	22 (10%)	24 (9%)	29 (9%)	17 (11%)
stage IIIB	2 (0%)	1 (1%)	1 (0%)	0 (0%)	2 (1%)	2 (1%)	0 (0%)
stage IIIC	27 (6%)	11 (10%)	16 (4%)	9 (4%)	18 (7%)	17 (5%)	10 (6%)
stage IV	21 (4%)	9 (8%)	12 (3%)	11 (5%)	10 (4%)	16 (5%)	5 (3%)
unknown	2 (0%)	1 (1%)	1 (0%)	1 (0%)	1 (0%)	1 (0%)	1 (1%)
			p = 0.038 sign.*		p = 0.352 n.s.*		p = 0.437 n.s.*
Chemotherapie							
yes	291 (61%)	65 (59%)	226 (61%)	149 (68%)	142 (55%)	210 (64%)	81 (53%)
no	145 (30%)	36 (33%)	109 (29%)	52 (24%)	93 (36%)	84 (26%)	61 (40%)
unknown	44 (9%)	9 (8%)	35 (9%)	19 (9%)	25 (10%)	32 (10%)	12 (8%)
			*p = 0.7 n.s.*		***p < 0.01 sign.***		***p < 0.009 sign.***
Estrogen							
negative	57 (12%)	14 (13%)	43 (12%)	35 (16%)	22 (8%)	46 (14%)	11 (7%)
positive	419 (87%)	94 (85%)	325 (88%)	183 (83%)	236 (91%)	278 (85%)	141 (92%)
unknown	4 (1%)	2 (2%)	2 (1%)	2 (1%)	2 (1%)	2 (1%)	2 (1%)
			*p = 0.4 n.s.*		***p = 0.04 sign.***		*p = 0.06 n.s.*
Progesteron							
negative	63 (13%)	15 (14%)	48 (13%)	40 (18%)	23 (9%)	52 (16%)	11 (7%)
positive	413 (86%)	93 (85%)	320 (86%)	178 (81%)	235 (90%)	272 (83%)	141 (92%)
unknown	4 (1%)	2 (2%)	2 (1%)	2 (1%)	2 (1%)	2 (1%)	2 (1%)
			*p = 0.4 n.s.*		***p = 0.01 sign.***		***p = 0.023 sign.***
HER2-Status							
negative	362 (75%)	82 (75%)	280 (76%)	158 (72%)	204 (78%)	236 (72%)	126 (82%)
positive	107 (22%)	26 (24%)	81 (22%)	57 (26%)	50 (19%)	84 (26%)	23 (15%)
unknown	11 (2%)	2 (2%)	9 (2%)	5 (2%)	6 (2%)	6 (2%)	5 (3%)
			*p = 0.8 n.s.*		*p = 0.2 n.s.*		***p = 0.022 sign.***

= I vs. II vs. III vs. IV

According to national AGO guidelines, chemotherapy was administered in a high frequency in cases with elevated uPA and PAI-1 levels (*P* < 0.01 and *P* < 0.009). Nevertheless, there was a considerable number of patients who did not receive chemotherapy despite elevated protease levels (52 and 81 patients).

### Correlation of proteases and dCK+ cells with survival

#### Complete cohort

Analyzing the whole cohort (*n* = 480), we identified an association between uPA, grading, and dCK+ cells with dCK+ rates of 18.8% vs 27.7% in uPA-negative and -positive cases, respectively (*P* = 0.028; *BH*_*ST*_ *0.013*). There was a trend toward reduced overall survival in patients with high PAI-1 levels with mean overall survival times of 112 months (CI: 104–119 months) versus 118 months (CI: 111–126 months); (*P* = 0.128 *BH*_*ST*_ *0.006*). Neither uPA- nor dCK+ cell analysis was prognostic with identical Kaplan-Meier curves for positive and negative cases.

#### Cases with chemotherapy

In cases with administered chemotherapy (*N* = 291), neither the proteases nor dCK+ cells were prognostic. Also, cases with triple positivity of uPA, PAI-1 and dCK+ cells did not show a different outcome compared to the other constellations.

#### Node-negative cases

**Fig. 2:**
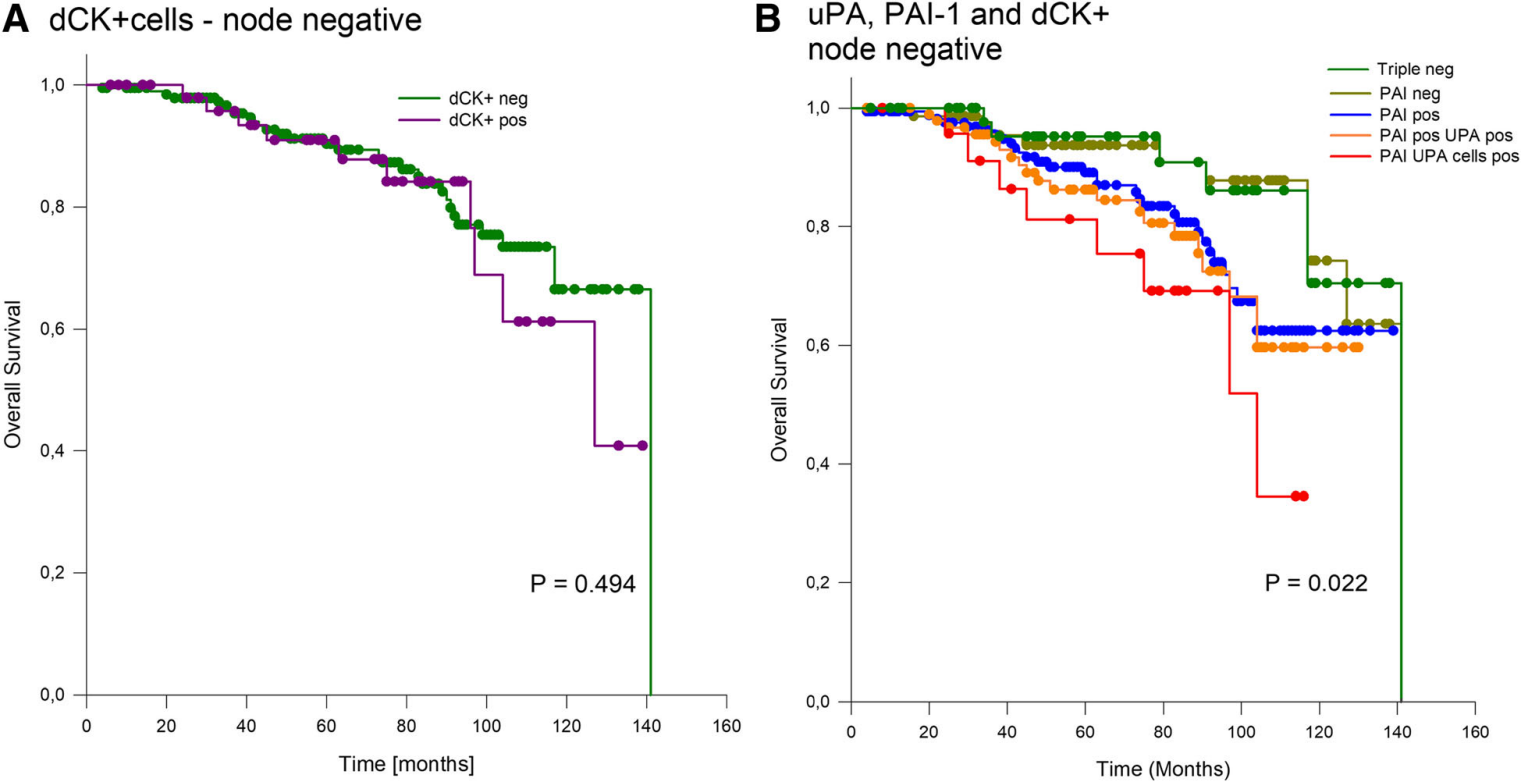
Overall survival in node-negative cases. **a**) Outcome dependent on the occurrence of disseminated cytokeratin-positive cells (dCK+). **b**) Outcome dependent on the occurrence of dCK+ cells and positivity of the proteases uPA and/or PAI-1 positivity is defined as tissue level above the cut-off. Note: *BH*_*ST*_ *= 0.01*

This subgroup comprised 249 cases. Again, a trend towards impaired outcome was seen in cases with PAI-1 positivity with mean overall survival times of 114 months (CI: 111–126 months) and 126 months (CI: 117–135 months) (*P* = 0.081 *BH*_*ST*_ *0.007*). A marginally significantly different overall survival was found in uPA-negative versus -positive cases with mean survival times of 123 months (CI: 116–130 months) and 108 months (CI: 100–116 months) (*P* = 0.065 *BH*_*ST*_ *0.008*). The identification of dCK+ cells was not prognostic (Fig. 2a). However, a significant difference, was found when comparing triple negative cases versus PAI-1-negative versus PAI-1-positive cases with uPA- and PAI-1- positivity (double positive) versus uPA-, PAI-1- and dCK+ cell positivity (triple-positive) (*P* = 0.022 BHST 0.01) with mean survival times of 127 months (Cl: 116–138 months), 126 months (CI: 117–135 months) (*P* = 0.045 BHST 0.01), 107 months (CI: 98–116 months), 115 months (CI: 108–122 months), and 90 months (CI: 75–105 months) (Fig. 2b). Pairwise Multiple Comparison Procedures (Holm-Sidak method) revealed significant differences between triple positive cases and PAI-negative cases (*P* = 0.014) and triple negative cases (*P* = 0.036). All other combinations, especially the comparison between double and triple positive cases were not significant.

#### Node-negative cases without chemotherapy

**Fig. 3:**
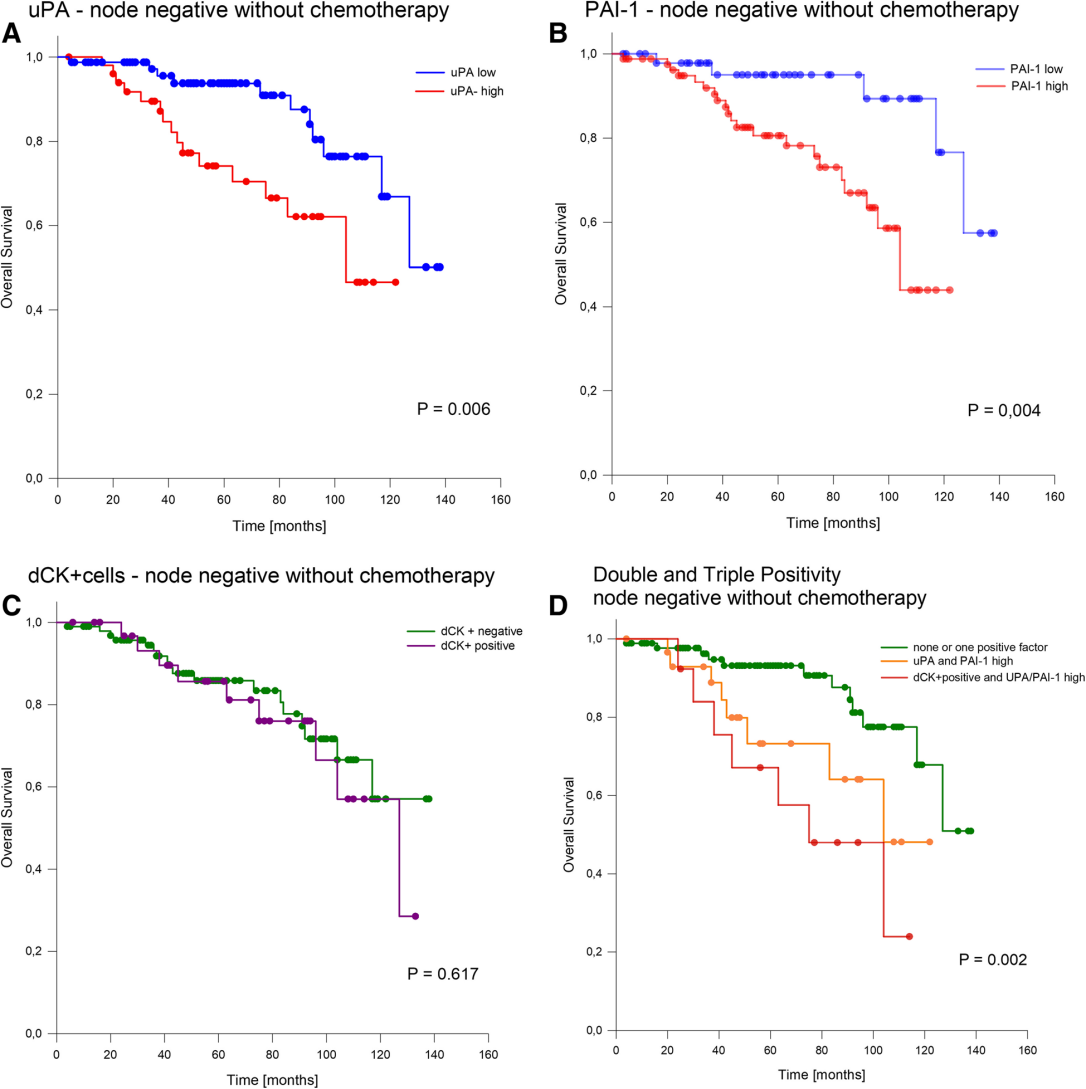
Overall survival in node-negative cases without chemotherapy. **a**) Outcome dependent on the tissue level of uPA *BH*_*ST*_ *= 0.017*. **b**) Outcome dependent on the tissue level of PAI-1 *BH*_*ST*_ *= 0.025*. **c**) Outcome dependent on the occurrence of disseminated cytokeratin positive cells (dCK+). **d**) Outcome dependent on the occurrence of dCK+ cells and positivity of the proteases uPA and PAI-1 *BH*_*ST*_ *= 0.05*

In locally limited tumors without nodal involvement and without known administration of adjuvant chemotherapy, both proteases were highly significant prognostic factors for overall survival (uPA: positive vs negative 91 months (CI: 78–103 months) vs 119 months (CI: 108–129 months); *P* = 0.006 (*BH*_*ST*_ 0.017) (Fig. 3a); (PAI-1: positive vs negative 94 months (CI: 84–103 months) vs 125 months (CI: 114–136 months); *P* = 0.004 *BH*_*ST*_ 0.025 (Fig. 3b). Disseminated CK+ cells alone were not found to be prognostic in this subgroup (dCK+ cells: positive vs negative 90 months (CI: 89–120 months) vs 112 months (CI: 101–122 months); *P* = 0.617 (Fig. 3c). However, triple-positive (PAI-1, uPA, dCK+ cells) cases show a significantly (*P* = 0.002 *BH*_*ST*_ *0.05*) worse outcome compared to cases with less than three positive factors with mean overall survival times of 76 months (CI: 56–98 months) vs 114 (CI: 105–123 months) (Fig. 3d). Pairwise multiple comparison procedures (Holm-Sidak method) revealed only a significant difference between the groups triple positive and one or none positive marker (*P* = 0.001), all other combinations were not significantly different. The survival was also shorter compared to cases with elevation of both proteases. However, this difference did not reach significance. Including PAI-1, uPA, dCK+ cells, T-stage, and grade into a multivariate analysis revealed T-stage (HR: 3.4 CI: 1.6–7.2) and triple positivity (HR: 9.3 CI: 1.1–75) as independent prognostic factors.

## Discussion

In this study, we retrospectively evaluated the relationship between and the potential interaction of uPA and PAI-1, which are two members of the plasminogen/plasmin system, and the occurrence of dCK + cells in in a cohort of 481 breast cancer cases. uPA and PAI-1 testing for therapy decisions in early breast cancer has been recommended for its routine use in early-stage invasive breast cancer by the American Society of Clinical Oncology [7] as well as the German AGO guidelines [14]. There are several approaches to evaluate the different factors of the plasmin/plasminogen system. These include immunohistochemical, ELISA- and reverse transcription polymerase chain reaction (RT-PCR) techniques. In the context of cancer, plasma and tumor tissue as well as tumor cells can be evaluated. For this study, a commercial ELISA test that found broad clinical acceptance analyzing fresh tumor tissue was used [15, 16]. This guaranteed a standardized evaluation. Despite this, the adherence to adjuvant chemotherapy in cases of elevated proteinases was low, with approximately 25% cases without adjuvant therapy in the high-risk groups (Table 1). Recently, we and others evaluated the effect of proteinases and multigene-assay testing on therapy decisions and concordantly found a lower impact of proteinase testing [17, 18]. We have no data elucidating the reason for that. One reason might be that the proportion of high-risk cases is rather high, and clinicians may be concerned that the risks due to adjuvant therapy might be higher than the acceptable benefits.

For the evaluation of circulating tumor cells, many different techniques have been developed. This concerns techniques of detection as well as the evaluated compartment of peripheral blood versus bone marrow. We used immunocytochemistry to detect cytokeratin 18-positive epithelial cells [10]. The technique of immunohistochemistry (CK18) based was well established in our laboratory and revealed reliable results [9, 19]. Using the same technique in colon specimens, we also detected positive cells in benign cases with diverticulitis [20]. Therefore, we think it is appropriate to avoid referring to these cells as tumor cells instead of dCK+ cells. Twenty-three percent of the cases in our collective were dCK+. This is a slightly lower rate compared to the pooled analysis including 4703 patients with a positivity rate of 30.6%. The positivity rate in this study, however, differed considerably between the contributing centers from 12.4 to 43.9%. Because of the availability of new methods and generally easier access, more recently performed studies have focused on CTC detection in peripheral blood. The detection rates in peripheral blood using modern techniques, such as the CellSearch™ system, RT-PCR, or immunofluorescence staining [8, 21], and evaluation of bone marrow and peripheral blood have yielded conflicting results. Schindlbeck et al. found comparable results when analyzing samples of peripheral blood and bone marrow [22]. Molloy et al. reported an additional adverse prognostic effect in cases in which tumor cells in both compartments were detected [23]. Only a weak concordance between blood and bone marrow samples was reported by Fehm et al. [24].

The plasminogen/plasmin system plays a crucial role in the degradation of several proteins, including fibrin, which leads to the dissolution of fibrin clots. Through cleavage, uPA activates plasminogen to plasmin, and PAI-1 is the physiological inhibitor in this context. Next to its function in fibrinolysis, this system is of high importance for the process of wound healing, which is the next step of repair after an injury. Here PAI-1 is not just an inhibitor of uPA, but an active player with distinct functions [25]. At least to some extent, cancer progression can be understood as a misdirected form of wound healing processes. The ability of uPA to degrade extracellular matrix is believed to facilitate tumor cell migration [26, 27]. Therefore, we assumed an association between uPA tissue levels and the occurrence of dCK+ cells. Indeed, we have identified such a relationship with a significantly higher rate (18.8% vs 27.7%) of dCK+ positivity in uPA-positive cases (*P* = 0.028). Such an effect was also found for PAI-1, but it was considerably lower (20.1% vs 24.2%) and did not reach significance. During wound healing, PAI-1 is expressed at the edge of an injury, stimulating attachment-detachment-reattachment processes [25, 28]. For detachment and cell migration, interaction between PAI-1 and uPA and its receptor uPAR and the lipoprotein receptor-related protein 1 (LRP1) is needed. This is a complex system, and our data may indicate that uPA plays an especially important role in the detachment of tumor cells and tumor migration. To our knowledge, only a few studies have addressed the topic of interaction between uPA/aPAR or PAI-1 with circulating tumor cells [29–31]. Mego et al. and Thomas et al. reported in concordance to our results associations between uPA and the occurrence of circulating tumor cells [29, 30]. Meng et al. found an association between HER2-postivitiy and uPA-expression on circulating tumor cells [31]. We found a trend toward a higher HER2-positivity in uPA-positive cases and a significant association with PAI-1 positivity (Table 1). Association with circulating tumor cells and HER2 indicate an adverse effect. The prognostic significance of uPA/PAI in tumor tissue has been confirmed in several studies [16, 32, 33]. However, we emphasize that it was not the main goal of our study to re-evaluate the prognostic relevance of uPA/PAI-1. We therefore evaluated uPA and PAI-1 separately. In our case series, we found a clear trend toward poor overall survival in PAI-1-positive cases when we analyzed the whole collective. In the subgroup of node-negative cases, PAI-1 (*P* = 0.081) and uPA (P = 0,031) showed prognostic relevance regarding overall survival. This prognostic effect was much stronger in the subgroup of node-negative cases without chemotherapy which is in accordance with the literature [15]. This is also in agreement with prior evidence [16, 34] indicating that patients in early breast cancer stages and those with elevated proteases benefit from adjuvant chemotherapy. The presence of dCK+ cells was associated with nodal status and by trend with tumor size. However, it was not prognostic concerning overall survival regardless of nodal status and application of adjuvant chemotherapy. This is in contrast to data from the literature using the same method of cell detection [9, 19, 35]. The main difference compared to the most previously published studies is the choice of endpoint. Because of the retrospective design, we have chosen overall survival, while many other used disease-free or tumor-specific survival, which might be more precise and therefore ideal for a prospective approach. However, we possibly identified an additive effect when dCK+ cells were identified in cases with uPA/PAI-1 positivity. This finding must be considered with caution because the difference between double and triple positive cases lacked significance. In node-negative cases without chemotherapy, triple positivity (uPA+; PAI-1+, dCK+) and T-stage were the only independent prognostic factors. This could indicate that uPA and PAI-1 not only facilitate detachment and migration of tumor cells but may also help those cells to persist within the bloodstream and bone marrow and to develop distant metastases. PAI-1 promotes angiopoesis and inhibits p53-induced apoptosis [36], two major hallmarks of cancer. Activation of invasion, a third hallmark, has been mentioned above [5]. Fibrin is believed to protect cancer cells from immune surveillance, which displays the fourth hallmark. Because PAI-1 inhibits fibrinolysis, this is very likely to also promote metastases formation [27, 37]. Additionally, uPA/uPAR are mitogenic which is the 5th hallmark [27]. This broad and very complex interaction between uPA, PAI-1, and dCK+ cells could explain the observed additive and maybe even synergistic effects of those three factors regarding outcomes of patients in early breast cancer stages. However, this hypothesis has not been proven by the data of this study and requires further evaluation.

This study is limited by its retrospective design. In this context, the results of the bone marrow evaluation could not be re-evaluated, and we do not have data concerning the interobserver agreement of the two investigators. Being unable to control and/or monitor the different factors that may influence such a complex system makes it difficult to draw definitive conclusions. Although our complete collective comprises a relatively large number of patients with a considerable follow-up, the need to analyze subgroups makes this collective still rather small. A further limitation is the missing availability of further biomaterial for additional analyses like co-expression of different markers on dCK+ cells. Despite these limitations this study could serve as a basis of further investigations employing modern detection methods combined with single cell analyses.

## Conclusions

Our results indicate a potential biological interaction between the protease uPA, its inhibitor PAI-1, and dCK+ cells and an independent prognostic effect. uPA and PAI-1 were prognostic in the subgroup of node-negative breast cancer patients without chemotherapy. The adverse effect of an elevated proteolytic system may have been counteracted by adjuvant chemotherapy. Our data further underline the importance of the hemostatic system for tumor progression. However, this hypothesis has not been proven by the data of this study and requires further evaluation.

## Data Availability

Raw data are available from an open repository: https://figshare.com/articles/uPA_PAI1_dTC_Maerkl2019_openData_xlsx/8285651

## Abbreviations

AGO: Arbeitsgemeinschaft Gynäkologische Onkologie
APAAP: Alkaline phosphatase anti-alkaline phosphatase
BHST: Bonferroni-Holm significance treshold
CI: 95% confidence interval
CK: Cytokeratin
CTC: Circulating tumor cells
dCK +: Disseminating cytokeratin positive
ELISA: Enzyme-linked immune sorbent assay
FFPE: Formalin-fixed paraffin-embedded
H2SO4: Sulfuric acid
HER2: Human epidermal growth factor receptor 2
HR: Hazart ratio
LRP1: Lipoprotein receptor-related protein 1
N: Number
P: Probability
PAI: Plasminogen activator inhibitor
RT-PCR: Reverse transcription polymerase chain reaction
T-stage: Tumor stage
uPA: Urokinase-type plasminogen activator
uPAR: Urokinase-type plasminogen activator receptor
